# The Medical Remembrancer, or Book of Emergencies; in Which Are Concisely Pointed out the Immediate Remedies to Be Adopted in the First Moments of Danger from Poisoning, Drowning, Apoplexy, Burns and Other Accidents

**Published:** 1845-08

**Authors:** 


*The Medical Remembrancer, or Book of Emergencies ; in which are concisely pointed out the immediate remedies to be adopted in the first moments of danger from poisoning, drowning, apoplexy, burns and other accidents. With the tests for the principal poisons, and other useful information.* By Edward B. L. Shaw, M. R. C. S., & L. A. S. 12mo. pp. 112 ; Samuel S. and William Wood, New York, 1845.

We have given the title page of this little work at length, because it is descriptive of its contents. It is rare to meet with so small a book comprising so much valuable information, or one containing so little that is objectionable. By the young practitioner, especially in the country, it will be found a valuable prompter in many trying situations.

